# Silver Nanoparticles-Functionalized Textile against SARS-CoV-2: Antiviral Activity of the Capping Oleylamine Molecule

**DOI:** 10.1021/acsami.4c15289

**Published:** 2025-01-14

**Authors:** Tamyres Bernardo de Souza, Alice S. Rosa, Pamella Constantino-Teles, Vivian Neuza S. Ferreira, Braulio S. Archanjo, Carlos A. G. Soares, Paulo H. S. Picciani, Rafael A. Allão Cassaro, Milene Dias Miranda, Giordano Poneti

**Affiliations:** † Instituto de Química, Universidade Federal do Rio de Janeiro, Rio de Janeiro 21941-909, Brazil; ‡ Laboratory of Morphology and Virus Morphogenesis, Oswaldo Cruz Institute, Fiocruz, Avenida Brasil, Rio de Janeiro 21041-250, Brazil; § Programa de pós-graduação em Biologia Celular e Molecular, Instituto Oswaldo Cruz, Fundação Oswaldo Cruz, Rio de Janeiro 21041-250, Brazil; ∥ Materials Metrology Division, National Institute of Metrology, Quality, and Technology, Duque de Caxias, Rio de Janeiro 25250-020, Brazil; ⊥ Departamento de Genética, Universidade Federal do Rio de Janeiro, Rio de Janeiro 21941-617, Brazil; # Instituto de Macromoléculas Professora Eloisa Mano, Universidade Federal do Rio de Janeiro, Rio de Janeiro 21941-598, Brazil; ∇ Dipartimento di Scienze Ecologiche e Biologiche, Università degli Studi della Tuscia, Largo dell’Università, Viterbo 01100, Italy

**Keywords:** SARS-CoV-2, silver nanoparticle, oleylamine, capping molecule, decontaminant agent, antiviral activity

## Abstract

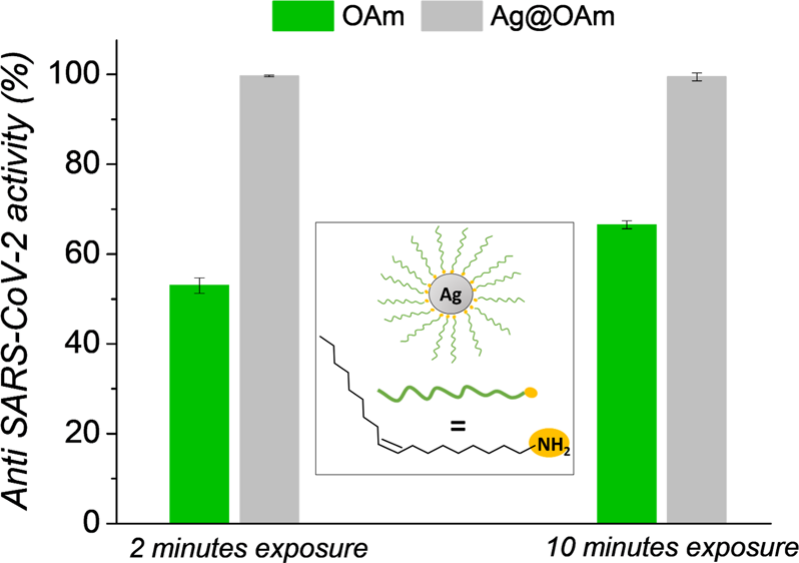

COVID-19 disease, triggered by SARS-CoV-2 virus infection, has led to more than 7.0 million deaths worldwide, with a significant fraction of recovered infected people reporting postviral symptoms. Smart surfaces functionalized with nanoparticles are a powerful tool to inactivate the virus and prevent the further spreading of the disease. Literature reports usually focus on the role of nanomaterial composition and size dispersion in evaluating their efficacy against SARS-CoV-2. Here, the anti-SARS-CoV-2 activity of oleylamine (OAm) used as a capping agent of silver nanoparticles is quantified for the first time. Spherical hydrophobic nanoparticles with 8 ± 2 nm diameter were prepared and characterized by Fourier transform infrared, dynamic light scattering, and transmission electron microscopy techniques. Biological assays showed that microgram amounts of nanoparticles, deposited on nonwoven textile obtained from surgical masks, efficiently inactivated up to 99.6(2)% of the virus with just 2 min of exposure. The virucidal activity of the corresponding amount of free OAm has been determined as well, reaching up to 67(1)% of activity for an exposure time of 10 min. Inductively coupled plasma optical emission spectrometry results pointed out a low leaching out of the nanoparticles in contact with water or culture medium. All in all, these results propose the capping molecules as an important chemical variable to be taken into account in the design of fast, efficient, and long-lasting anti-SARS-CoV-2 coatings.

## 1 Introduction

Severe acute respiratory syndrome Coronavirus 2 (SARS-CoV-2) is a virus that emerged in December 2019 in China and gave rise to Coronavirus disease 2019, also known as COVID-19. Due to its high transmissibility, this virus quickly spread across the world population giving rise to a global pandemic. According to the World Health Organization (WHO), the virus infected about 775 million people and more than 7.0 million people died from complications of this disease.^1^ In addition to that, the long-term post-COVID-19 effects led to heart or neurologic persistent damage, fatigue, breathing difficulty, memory, and thinking problems whose recovery time is still to be determined.^2^ Its main form of transmission is by air as droplets of saliva are dispersed and remain infective for up to 3 h depending on the viral load and strain.^3^ However, contaminated surfaces are an additional threat, the virus being active for up to 48 h, depending on the surface.^3^ Currently, several forms of surface decontamination are quite effective against SARS-CoV-2 such as 70% ethanol or 2-propanol, aqueous solutions of sodium hypochlorite 0.1%, and ultraviolet radiation, but these sources require frequent application of decontaminant agent leading to a relatively large cost and in some cases do not meet the sustainability features required for a long-term antiviral policy. The development of efficient, long-lasting decontaminant agents and active smart surfaces is a global issue.

Over the years, the development of nanomaterials for biomedical applications has intensified. While gold and iron oxide nanoparticles (NPs) have shown promise as sensors^4^ and therapeutic applications,^5^ respectively, silver-based nanomaterials are known to be highly active in the fight against bacteria,^6^ fungi,^7^ and viruses.^8,9^ In the context of the COVID-19 pandemic, various nanomaterials have been investigated against SARS-CoV-2, including titanium dioxide^10,11^_,_ copper oxide,^12,13^ iron oxide NPs (Fe_2_O_3_ and Fe_3_O_4_),^14^ and graphene.^15^ Ag-based materials have shown virucidal activity in the form of composites^16−18^ or nanostructured materials.^19−21^ Silver nanoparticles (Ag NPs) could effectively inhibit the activity of SARS-CoV-2, and different surface modifications and particle sizes conferred different virucidal effects.^20^ As such, their implementation as an antiviral coating in textiles has been published^22−24^ and patented.^25^

In the process of nanomaterial optimization with anti-SARS-CoV-2 properties, the main research efforts have scanned for different materials, sizes,^19^ and morphologies.^26^ Despite determining several physicochemical properties of nanomaterials, like solubility, contact angle, and stability in solution, the surfactants coating the nanostructured core of the materials have been less looked upon as a chemical variable for increasing their antiviral activity.^20^ In 2021, a data-mining investigation looked at the best candidates, among surfactants, for docking and inactivating SARS-CoV-2,^27^ but only one experimental report described the role of zeta potential in inactivating SARS-CoV-2, showing that positively charged surface layers are more effective than negative ones.^20^

Here, we aim to quantitatively describe the activity of the NP capping agent, oleylamine (OAm), against SARS-CoV-2. The choice of OAm as a ligand is based on its structural resemblance to linoleic acid, a fatty acid that has been implicated in stabilizing the locked conformation of the SARS-CoV-2 spike protein.^28,29^ This stabilization reduces the likelihood of the spike protein to interact with the angiotensin-converting enzyme 2 (ACE2) receptor, the primary entry point for the virus into human cells.^30^ Both OAm and linoleic acid, in fact, share a long aliphatic chain consisting of 18 carbon atoms and a *cis*-double bond at carbon 9, and linoleic acid features an additional *cis*-double bond at carbon 12. Despite this slight structural difference, OAm offers a significant advantage in terms of lower cost, making it a more feasible and economically viable option for potential SARS-CoV-2 inactivation strategies. Studies have also shown that the hydrophobic nature of these molecules can play a key role in disrupting viral lipid membranes, further supporting the exploration of OAm as a practical antiviral agent with a virucidal effect.^28^ Indeed, silver NP coated with OAm deposited on top of surgical masks displayed activity against the HCoV-229E virus,^21^ but their antiviral activity against the SARS-CoV-2 virus as well as the contribution of the ligand have not been investigated yet.

In this contribution, OAm-coated Ag NPs (Ag@OAm), deposited on the textile of surgical masks made of nonwoven fabric with three layers of protection, inactivated up to 99.6(2)% of the SARS-CoV-2 virus in 2 min, while the corresponding amount of OAm ligand showed 53(2)% of antiviral activity. Both the antiviral activity of OAm and Ag@OAm increased with the amount of material and exposure time, reaching up to 67(1) and 100(1)%, respectively, for 10 min. Thus, this study proposes OAm-coated nanomaterials as a sustainable alternative for the preparation of personal protective equipment (disposable lab coats and caps) and different solid matrices such as hospital bed sheets and textiles in general.

## 2 Experimental Section

### 2.1 Materials

Unless indicated otherwise, all manipulations were performed under aerobic conditions using materials as received. Toluene (99%, Tedia), ethanol (Bio-Grade), *n*-hexane (95%, Tedia), OAm (technical grade, 70%, Sigma-Aldrich), oleic acid (OAc, technical grade, 90%, Sigma-Aldrich), and AgNO_3_ (Sigma-Aldrich) were used as received. MTT salt (3-[4,5-dimethylthiazol-2-yl]-2,5 diphenyl tetrazolium bromide, Sigma-Aldrich), DMEM (Dulbecco’s modified Eagle’s medium; Gibco, Waltham, MA, EUA/Life Technologies, Grand Island, NY, EUA), FBS (fetal bovine serum, Gibco, South American), CMC (carboxymethyl cellulose solution, Sigma-Aldrich), and SDS (sodium dodecyl sulfate, Sigma-Aldrich) were used in assays.

### 2.2 Synthesis of Ag@OAc

Ag NPs coated with OAc (Ag@OAc) were prepared with a slight modification of the procedure reported by Hyeon and co-workers.^31^ To 13.5 mL of OAc and 1.5 mL of OAm was added 5.18 g of silver nitrate under stirring. The reaction medium was purged using vacuum/argon at 70 °C for 1.5 h. The yellow solution was heated up to 180 °C at the heating rate of 10 °C/min under an argon atmosphere. The temperature was kept at 180 °C for 2 min, leading to a brown solution. After cooling to room temperature, the purification of the NPs was carried out by adding 150 mL of ethanol to the reaction medium, followed by centrifugation at 4000 rpm for 10 min. The supernatant was discarded, and the solid was suspended in 30 mL of toluene. This purification procedure was repeated twice.

### 2.3 Ligand Exchange for OAm (Ag@OAm)

For ligand exchange, a forty-fold excess of OAm relative to the mass of silver (determined by inductively coupled plasma optical emission spectrometry, ICP-OES) was added, and the toluene solution was maintained for 24 h under constant stirring at room temperature. The NPs were precipitated by adding ethanol, centrifuged at 4000 rpm, and resuspended with *n*-hexane. The suspension was stocked at 4 °C in *n*-hexane.

### 2.4 Characterization

The NPs were characterized by UV–vis spectroscopy at room temperature in solution on a Shimadzu UV-2600 spectrometer. IR spectra were recorded using a Nicolet Magna FT-IR spectrometer, model IR-760, in the range 400–4000 cm^–1^ using a KBr pellet. The samples used in the leaching tests were weighted using a PerkinElmer AD-4 autobalance. Thermogravimetric analysis (TGA) data were obtained using a Shimadzu DTG-60 and a Q500 analyzer from TA Instruments in the temperature range of 30–480 °C under an argon atmosphere at a heating rate of 10 °C min^–1^. ICP-OES data were obtained using a Spectro, model Arcos. The sample was dissolved in nitric acid using a digestion tube. Dynamic light scattering (DLS) was recorded using a Zetasize Malvern instrument in an *n-*hexane solution. Transmission electron microscopy (TEM) images were obtained using an FEI Tecnai Spirit microscope working at 120 kV. ImageJ software was used for measuring the particle diameters of the images obtained from TEM. Scanning electron microscopy (SEM) images were obtained with a FEI Company Magellan 400 microscope. Energy-dispersive X-ray spectroscopy (EDS) was performed using the Thermo Scientific Noran System 7 coupled to SEM equipment.

### 2.5 Impregnation of Ag@OAm and OAm on Textile

The source of textile was a nonwoven fabric with three layers of protection surgical masks. 0.6 cm diameter discs (mass around 2 mg) were cut from the mask and put in a 96-well plate, followed by exposure to ultraviolet light for 30 min at room temperature for decontamination. After that, different amounts of Ag@OAm were added from their *n*-hexane suspensions (2.9, 5.9, 11.8, 23.6, and 47.1 μg) using the drop-casting technique. The OAm samples were prepared in the same manner considering the same amount of ligand present in Ag@OAm (2.6, 5.3, 10.5, 21.1, and 42.1 μg). As a control, the solvent (*n*-hexane) was added to the discs, and all samples were left for 2 h at room temperature for complete evaporation of the solvent before analysis. The whole procedure of impregnation was performed in a laminar flow hood to avoid contamination.

### 2.6 Leaching Out Tests of Ag@OAm in Water and Culture Medium

Retainment of nanostructures in the hybrid nanomaterials is of paramount importance,^21,32^ especially considering the harmful nature of OAm. The test of leaching out was performed with the discs impregnated with an amount in the range of 150–230 μg of Ag@OAm. 2.5 mL of water was added and kept in contact for different times (2 min and 15 h). After this time, the water was removed, and its Ag content was evaluated using the ICP-OES technique, while the discs were dried overnight in the dark at room temperature. For the short contact time, this test was repeated three more times (washing cycles), and a visual inspection of the pictures of the discs before and after contact with water is shown in Figure S1. The test of leaching out was also performed using 600 μL of culture medium (DMEM high glucose, Gibco) instead of water. The exposure times were the same as those used in the anti-SARS-CoV-2 assays (2 or 10 min) (see below).

### 2.7 Anti-SARS-CoV-2 Assays

For anti-SARS-CoV-2 assays, 96-well plates containing Ag NPs impregnated on the textile and controls were prepared as previously described. SARS-CoV-2 virus (lineage B.1, GenBank #MT710714, SisGen AC58AE2) was stored at −80 °C and handled at the biosafety level 3 (BSL3) laboratory, in accordance with the World Health Organization guidelines.^33^ For these assays, parameters similar to those described in International Standard ISO 18184 “Textiles–Determination of antiviral activity of textile products”^34^ for assays with the influenza virus were used, which suggests exposure to 50 μL of a viral titer of 5.0 × 10^4^ PFU/mL (PFU, plaque-forming unit) for 30 min at 25 °C. The SARS-CoV-2 suspension for the antiviral assay was prepared in DMEM high glucose without FBS, 100 U/mL of penicillin, and 100 mg/mL of streptomycin (Gibco). A viral titer equivalent to an MOI (multiplicity of infection, ratio of infectious viral titer per cell) of 0.5 was used for this suspension. Since the wells for the viral infection challenge contained 2.0 × 10^4^ cells per well, we used 2.0 × 10^4^ PFU per well in a volume of 100 μL. 100 μL of this virus suspension was exposed to the impregnated textile with the materials to be tested or the controls. After 2 or 10 min at 25 °C, 50 μL of the virus suspension was collected (equivalent to 10^4^ PFU/50 μL or 2.0 × 10^5^ PFU/mL), and the viral particles were challenged to carry out their infection in a highly permissive cell model, Vero E6 cells (African green monkey kidney cells, ATCC CRL-1586). To this aim, 50 μL of the virus suspension postexposition of different conditions were thus transferred to a 96-well plate containing Vero E6 cells previously prepared (2.0 × 10^4^ cells/well). The plate was incubated for 1 h at 37 °C and 5% CO_2_. Then, the culture medium was exchanged with 100 μL of DMEM with 2% of FBS, and the plate was incubated for 24 h at 37 °C and 5% CO_2_. Then, the cell supernatants were collected for virus titration by the PFU assay (PFU/mL). In brief, 50 μL of supernatants were diluted in the medium (1:2, 1:4, 1:8, 1:16, 1:32, 1:64, 1:128, 1:256) and transferred to Vero E6 cell plates (2.0 × 10^4^ cells/well). Again, the plates were incubated for 1 h. After this time, 50 μL of CMC solution (DMEM-HG 10×, 2,4% de CMC, and 2% FBS) was added, and the plate was incubated for 72 h under the conditions previously mentioned. Then, 50 μL of 4% formalin solution was added to fixate the cells. Three hours later, the supernatant was discarded, and the plate was stained with 0.04% crystal violet solution for 30 min, and PFU was counted.^18^ These assays were performed in triplicate on two different days. The percentages of virus inhibition were calculated in relation to the infection control, i.e., the viral suspension without exposure to Ag@OAm, OAm, or the controls, which were subjected to the same temperature as all experiments.

### 2.8 Cytotoxicity Assay

The cell viability tests were performed to corroborate the scientific solidity of our antiviral tests, in particular, to evaluate the effect of the functionalized textiles on Vero E6 viability for all different experimental conditions employed (mass of Ag@OAm, OAm, or controls and exposure time). For this purpose, 100 μL/well of a solution containing DMEM without FBS, 100 U/mL of penicillin, and 100 mg/mL of streptomycin (Gibco) were added in the 96-well plate containing discs of textile previously impregnated with different amounts of Ag@OAm, OAm, or just vehicle (*n-*hexane) used as control. The culture medium was exposed to the material for 10 min (the maximum exposure time used in the antiviral assay). After this exposure time, 50 μL of supernatant was transferred to a 96-well plate containing Vero E6 cells (2.0 × 10^4^ cell/well). The cells were incubated for 1 h at 37 °C and 5% CO_2_. Then the supernatant was exchanged with 100 μL of DMEM with 2% of FBS, the plate was incubated for 24 h under the same conditions (as was done for antiviral assay), and cell viability was assessed using the MTT method following the manufacturer’s instructions. Briefly, 10 μL/well of MTT 5.0 mg/L solution was added, and the plate was incubated for 2 h, followed by the addition of SDS 10%. Then, the plate was incubated for 2 more hours. The spectrophotometric analysis was carried out at 570 nm for absorbance analysis.^35^

### 2.9 Statistical Analysis

One-way ANOVA with Dunnett’s post-test was used to analyze the significant difference in viability assay. Two-way ANOVA with Tukey post-test was used to determine the significant difference in anti-SARS-CoV-2 experiments. Three discs with the same amount of Ag@OAm or OAm or controls were used in two independent assays, and *p* < 0.05 was considered statistically significant. Asterisk (*) was used to show the difference in relation to *n-*hexane; *p ≤ 0.05, **p ≤ 0.01, *** or ^###^*p* ≤ 0.001. Data were analyzed using GraphPad Prism 10.1.0. In the text, the number between parentheses represents the standard deviation of the average value obtained from the antiviral assays.

## 3 Results and Discussion

As mentioned in the introduction, the choice of OAm stems from its structural similarity with linoleic acid, which has been proposed to bind to the SARS-CoV-2 spike protein, stabilizing its locked conformation, thus making it less prone to interact with the human ACE2 receptor.^28,29,36^ Furthermore, considering the hydrophobic nature of both OAm and linoleic acid, it is likely that their interaction with the viral lipid membrane contributes to their virucidal properties.^37^ Disruption of the viral envelope, which is crucial for virus stability and infectivity, may further enhance the antiviral effects, providing an additional layer of inhibition against SARS-CoV-2. Finally, the choice of the OAm ligand provides hydrophobicity to the NPs, making them less prone to being washed out by water from the functionalized textile.

### 3.1 Synthesis and Characterization of Ag@OAc and Ag@OAm

Silver nanoparticles Ag@OAc were synthesized following a modification of a previously reported procedure,^31^ consisting of using OAc as the solvent and OAm as the reducing agent at a high temperature under an inert atmosphere, followed by purification by centrifugation. The UV–vis spectrum of Ag@OAc is reported in Figure S2. A ligand exchange reaction was carried out at room temperature for 24 h using a 40-fold excess of OAm, yielding Ag@OAm. The ligand exchange from OAc to OAm is expected to ensure the stability of the NPs on the textile support since the virucidal tests were performed in an aqueous solution. In fact, the oleate molecules on the surface of the Ag@OAc NPs could be replaced by water molecules since both species have oxygen as the donor atom. These would lead to the removal of OAc from the surface of the NP and presumably decrease its hydrophobicity, favoring the leaching out process in water. According to hard and soft acids and bases Pearson’s principle,^38,39^ a stronger noble metal–ligand bond is expected when the metal is coordinated by atoms softer than oxygen, such as nitrogen. Replacement of OAc with OAm capping molecules is thus expected to increase the stability of the NPs toward the dissociation of the capping ligands.

A comparison between the infrared absorption spectra of Ag@OAm and Ag@OAc and the corresponding capping ligands is shown in Figures S3 and S4. For Ag@OAm, the broad band observed at 3437 cm^–1^ is attributed to N–H stretching,^40,41^ while the bands at 2920 and 2854 cm^–1^ refer to the asymmetric stretching of C–H bonds^40^ (Figure S3). When compared with the spectrum of Ag@OAc (Figure S4), the N–H stretching absorption is absent. On the other hand, Ag@OAc presents a strong band at 1710 cm^–1^ attributed to C=O stretching,^40,42^ absent in the Ag@OAm spectrum. These results point to an effective replacement of OAc by OAm on the surface of the Ag NPs.

**Figure 1:**
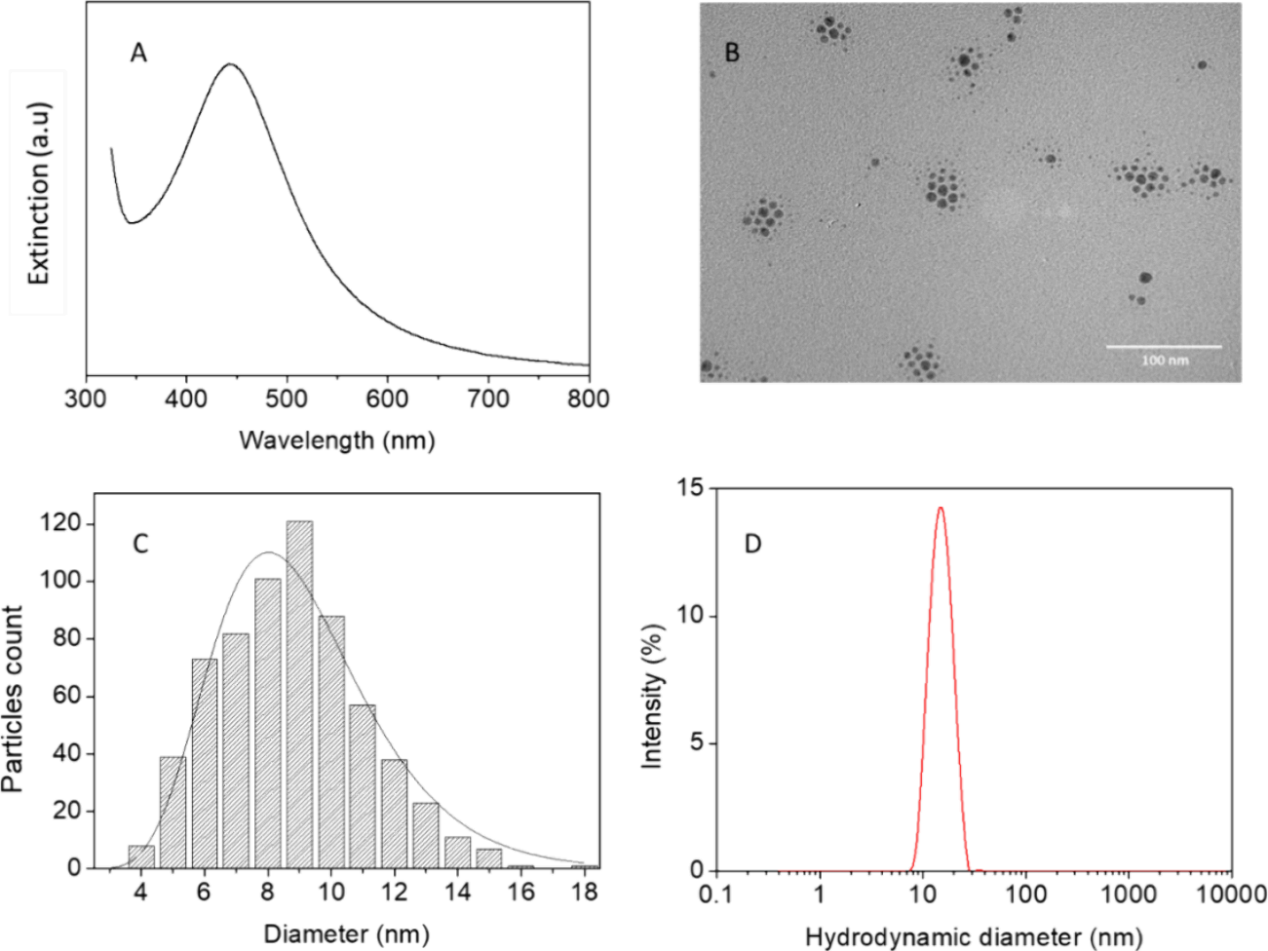
Ag@OAm characterization: UV–vis spectrum in *n*-hexane (A). Representative TEM image (B) and size distribution histogram with log-normal fit (solid line) (C). DLS in *n*-hexane (D).

The electronic spectrum of Ag@OAm (Figure 1A) displays a maximum of the extinction peak at 428 nm, characteristic of spherical Ag NPs obtained by the chemical method.^43^ TEM results (shown in Figure 1B,C) show that Ag@OAm are spherical with an average diameter of 8 ± 2 nm. The DLS (Figure 1D) analysis supports this result, indicating a hydrodynamic diameter of 16 nm.

The relative amounts of silver metal and organic ligand coated on the surface were determined by ICP-OES and TGA. The TGA profile displays two losses of mass, being attributed to the solvent weakly interacting with the surface of the NPs (25–60 °C) and to the loss of OAm in the 150–480 °C range. The second loss of mass is incomplete, indicating that higher temperatures should be reached for the total decomposition of OAm (Figure S5). Therefore, the expected overestimated amount of silver determined by TGA (14% of Ag) is not far from the value obtained by ICP, showing that silver corresponds to 11% of the total mass of the NPs. The masses of Ag@OAm used in the biological assays are reported in Table 1, along with the corresponding OAm and silver amounts, calculated on the basis of ICP results. The amounts of OAm and silver in the NPs are important parameters in rationalizing the antiviral tests (see below).

**Table 1:** Amounts of Ag@OAm, Ligand, and Silver Used in the Biological Tests

Ag@OAm mass (μg)	OAm mass (μg)	Ag mass (μg)
47.1	42.1	5.0
23.6	21.1	2.5
11.8	10.5	1.3
5.9	5.3	0.6
2.9	2.6	0.3

### 3.2 Impregnation of Ag@OAm and Free OAm on Textile

**Figure 2:**
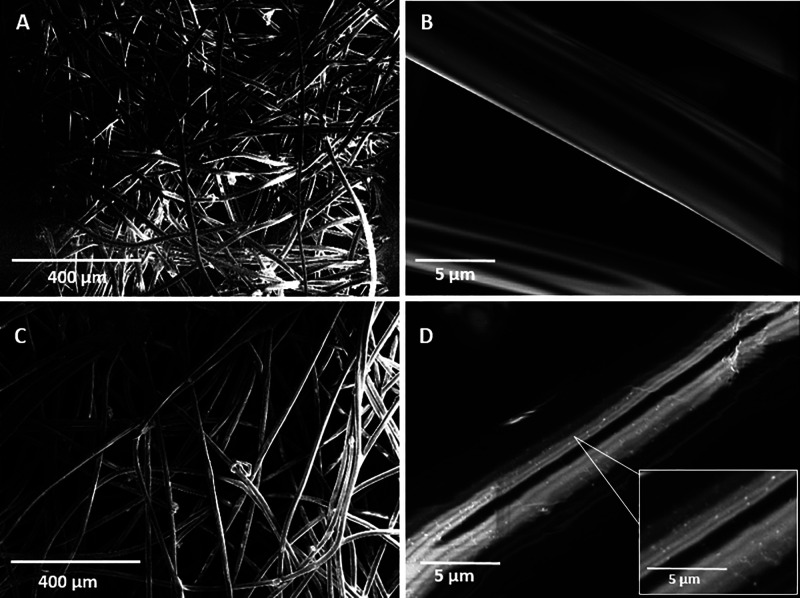
SEM images of bare (A, B) and Ag@OAm-functionalized textile (C, D) with zoom in the region of textile fibers (D).

The deposition of Ag@OAm, as well as free OAm, on textile has been carried out by drop-casting *n-*hexane suspensions of the samples at room temperature, followed by solvent evaporation. When compared with the bare, untreated textile (Figure 2A, B), SEM images reveal that the textile functionalized with Ag@OAm exhibits comparable roughness (Figure 2C) and dispersed spherical small Ag NPs on the surface of the material (Figure 2D). EDS analysis (shown in Figure S6) confirms the presence of silver on the surface of the functionalized textile.

Beyond affording the successful functionalization of the textiles, the hydrophobic OAm coating of the NPs hindered their affinity for water, leading to negligible leaching out of Ag@OAm. In particular, for short contact times with water (2 min), ICP-OES analysis of the supernatant as well as visual inspection of the discs (Figure S1) indicated that Ag@OAm did not leach out. The same experiments pointed out about 1% of leaching of the overall Ag content after 2 or 10 min exposure to the cell culture medium. Upon 15 h exposure time to pure water, however, ICP-OES showed that a small amount of Ag NPs (around 2% of the total amount added to the textile) had been washed out. All in all, this evidence confirms the potential of the composite material as a long-standing decontaminant agent.

### 3.3 Anti-SARS-CoV-2 Activity and Cytotoxicity Assay of Ag@OAm

**Figure 3:**
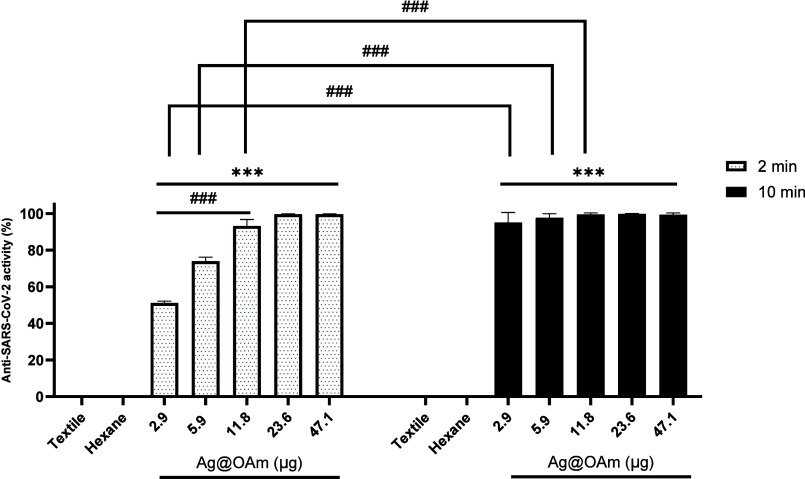
Anti-SARS-CoV-2 activity of different amounts of Ag@OAm impregnated on textile for exposure time of 2 min (left, white bars) or 10 min (right, black bars). Experimental controls: virus infection in pure textile and *n-*hexane added to the textile, followed by evaporation. Two-way ANOVA was used to analyze the statistical difference between the groups. (*) represents comparison with *n-*hexane, and (^#^) represents comparison among Ag@OAm amounts. *** or ^###^*p* ≤ 0.001. *n* = 6.

For biological assays, Vero E6 cells were used since these cells are permissive to SARS-CoV-2 replication and have ACE2 as a principal receptor for this virus infection.^44^ The assessment of the anti-SARS-CoV-2 activity of Ag@OAm was performed by exposing the viral suspension to the textile impregnated with different amounts of NPs for different times (2 or 10 min). After this exposure, the viral particles were challenged to infect Vero E6 cells. Viruses grown in these cells for 24 h were titrated by the PFU assay, revealing the clear antiviral properties of the Ag@OAm-treated textile (Figure 3).

The anti-SARS-CoV-2 activity was found to be dependent on the NP amount as well as on the duration of exposure to the functionalized textile. After 2 min of exposure, the sample containing the lowest amount of Ag@OAm NPs (2.9 μg) displayed an inactivation capability of 51.2(8)%, a percentage that rose to 74(2) and 93(3)% for 5.9 and 11.8 μg, respectively, and to 99.6(2)% for 23.6 and 47.1 μg of Ag@OAm. The data obtained after an incubation time of 2 min point out an increasing antiviral activity of the nanostructure with increasing amounts of Ag NPs. This result is in line with other studies reported in the literature, which show that larger amounts or higher concentrations of Ag NPs have a greater effect against other viruses.^9,45^ Additionally, the antiviral effect of Ag NPs on SARS-CoV-2 has already been demonstrated but with a virus title at least 2 logs lower than that used in this study.^20^

These results show that the optimal amount of Ag@OAm is 23.6 μg, being the one showing the highest activity in 2 min of exposure time. On the other hand, for the exposure time of 10 min, all impregnation conditions were capable of significantly inhibiting SARS-CoV-2 infection (more than 95% for all amounts of Ag@OAm, Figure 3). It must be stressed that the Ag@OAm system presented here is one of the most powerful Ag-based anti-SARS-CoV-2 agents described so far, since a very small amount of Ag NPs, approximately 2% of the overall mass of the functionalized textile, corresponding to 84 μg/cm^2^, was effective to inactivate the virus in a short period of time (2 min). While previous studies reported quantitative SARS-CoV-2 inactivation using similar concentrations of the active system in the final composite material,^16,46−49^ only a few of them^16,49,50^ used a viral exposure time of up to 2 min, which is central for the development of effective decontaminating agents. The majority of the literature reports of Ag-functionalized substrates with anti-SARS-CoV-2 activity displayed less efficient behavior in terms of time exposure to the virus or overall antiviral activity. Indeed, in this study a viral titer equivalent to 2.0 × 10^5^ PFU/mL per assay has been used, consisting of 4 times the titer value recommended by the International Standard ISO 18184 “Textiles—Determination of the antiviral activity of textile products.”^34^ Moreover, this viral titer was inactivated in one-third of the recommended exposure time. These experimental conditions point out the strong antiviral potential of the material investigated here.

**Figure 4:**
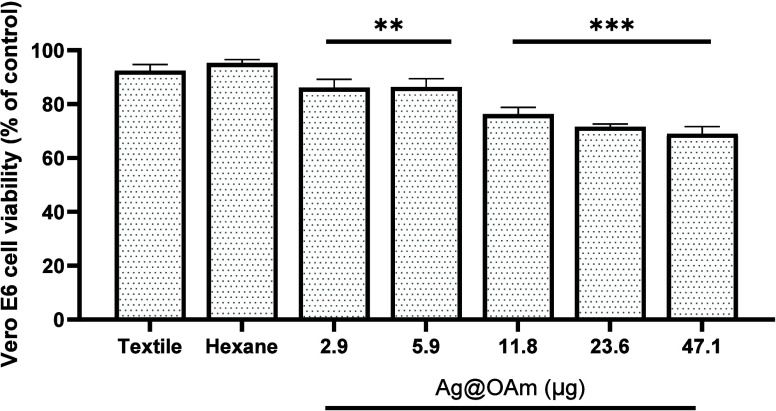
Cytotoxicity of Ag@OAm on the Vero E6 cells. The cells were exposed to the culture medium after contact with functionalized textile with different amounts of NPs (2.9, 5.9, 11.8, 23.6, 47.1 μg). The cell viability was assessed by MTT assay. *n*-hexane added to textile as well as the pure textile were used as experimental controls. Cells exposed just to culture medium were used as the cell control. The percentages of cell viability were calculated in relation to the cell control. *n* = 6. **p ≤ 0.01, and ***p ≤ 0.001.

The cytotoxicity assay was performed to assess whether any factor of the product being tested in the antiviral assay could promote cell death, and thus the observed inhibition of replication might simply be due to nonviable cells. To avoid this bias, we conducted the cytotoxicity assay in exactly the same way as the antiviral assay but without the addition of the viral isolate and evaluated the viability of the cells exposed to the supernatant that had been in contact with the textile functionalized with Ag@OAm or OAm. The products promoted low to moderate toxicity on the exposed cells: the cell viabilities were above 66, 73, and 83% for cells exposed to 47.1, 11.8, and 2.9 μg of Ag@OAm, respectively (Figure 4). In every sample tested, the cell viability was greater than 66%, confirming the feasibility of Ag@OAm as a bioactive material. All in all, the cell viability tests showed lower cytotoxicity of Ag@OAm with respect to free OAm (see Figure S7).

The mechanism of the virucidal effect of Ag NPs against the SARS-CoV-2 virus is still not well established in the literature. However, Ag NPs can act in different stages of viral replication against other viruses such as human immunodeficiency virus type 1 (HIV-1),^45,51^ adenovirus type 3 (Ad3),^52^ influenza A (H1N1),^53^ and hepatitis B.^54^ In the first stage, Ag NPs could damage the entire viral particle of adenovirus type 3 (Ad3),^52^ disrupt viral particle of influenza H1N1 virus,^53^ or act in the early stages of HIV replication by interacting with the protein on the surface of the virus, which prevents its fixation in the host cells.^45,51^ Other forms of inhibition are supposed to occur inside the virion by the interaction of the Ag NPs with the nucleocapsid and the genetic material.^52,54^ Since the hydrophobic Ag@OAm is physisorbed on textile, the results obtained in this study suggest that the NPs act against SARS-CoV-2 in the initial stages when the Ag NPs can bind to the protein S (spike) present on the virus, preventing the binding with the receptor in host cells, leading to subsequent inhibition of viral replication or damaging the virion particle. The leaching out test of Ag@OAm in the culture medium corroborates this suggestion since only a very small amount (1%) of the overall deposited NPs is released for both exposure periods of 2 and 10 min. Although this test indicated that the NPs may be found in the culture medium, albeit with a very small concentration, it is likely that the primary antiviral activity is virucidal, given that the anti-SARS-CoV-2 effect is time-dependent.

### 3.4 Quantitative Evaluation of the Antiviral Activity of the Free OAm Ligand

**Figure 5:**
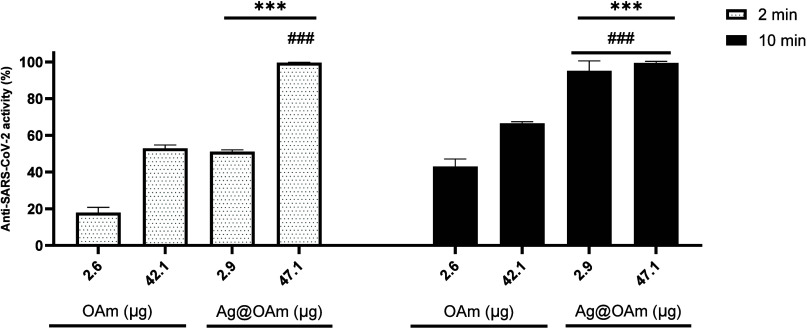
Comparison between the anti-SARS-CoV-2 activity of OAm and Ag@OAm for virus exposure times of 2 min (left, white bars) and 10 min (right, black bars). (*) and (#) represent the comparison with 2.6 and 42.1 μg of OAm, respectively. *n* = 6. *** or ###: *p* ≤ 0.001.

Figure 3 indicates that Ag@OAm is one of the fastest and most effective silver-based anti-SARS-CoV-2 agents reported to date, probably due to its virucidal activity, since the textile and the solvent used for the deposition of the NPs displayed negligible activity against the virus. A comparison between the activity of the two limiting amounts of Ag@OAm samples previously employed and the corresponding free OAm coverages for two different exposure times (2 or 10 min) to the virus is shown in Figure 5. The amount of OAm has been chosen to be 89% of the mass of the whole NP, according to the ICP-OES results.

For every analyzed sample, free OAm exhibited antiviral activities comparable to those shown by Ag@OAm. This activity increases with the amount of material deposited and with the exposure time to the viral suspension. Indeed, 2.6 μg of the OAm sample showed 18(3)% activity for a 2 min exposure time, while the heavier sample (42.1 μg) showed 53(2)%. In the analogous analysis in 10 min, these antiviral activities increased to 43(4) and 67(1)%.

These results suggest that the OAm coating of the silver nanostructures plays a relevant role in determining their anti-SARS-CoV-2 properties. It must be stressed that a detailed rationalization of these results would require additional experimental and *in silico* investigations. That said, previous molecular dynamics simulations^29^ showed the tendency of the linoleate anion to lock the SARS-CoV-2 spike protein in its folded conformation, thus reducing its affinity to the ACE2 human receptor. On the basis of this study, we hypothesize that the hydrocarbon tails of the OAm component, structurally related to the aliphatic backbone of the linoleate anion, change the membrane architecture/landscape and viral protein function for cellular infection (recruitment/entry). In addition to that, the OAm coverage also gives the Ag NPs the hydrophobicity required to interact with nonwoven textiles, at the same time reducing leaching on contact with water, as desired for a long-lasting decontaminant agent. All in all, this study shows that the surface layer of silver nanostructures is of paramount importance in the design of innovative textiles aimed at reducing the transmission of SARS-CoV-2.

## 4 Conclusions

Here, a simple drop-casting method was used to functionalize textile made of nonwoven fabric with three layers of protection with OAm-coated Ag NPs (Ag@OAm). The anti-SARS-CoV-2 study demonstrates that few micrograms of the NPs deposited are effective in combating the virus even for brief exposure times (2 min), making the functionalized textile used in this study one of the most powerful silver-based anti-SARS-CoV-2 agents reported to date. A comparative analysis with the free OAm molecule reveals that the antiviral activity of the Ag@OAm nanostructure is higher but indeed comparable to the one measured for its capping agent alone. Additionally, the hydrophobic nature of the Ag@OAm samples makes them ideal for coating surfaces that will not be washed out by water, which is essential for effective and durable decontaminant agents. Moreover, employing cheap coating molecules like OAm is crucial for the economically sustainable large-scale production of smart textiles with antiviral properties.

To conclude, this study marks the first quantitative analysis of the anti-SARS-CoV-2 activity of the OAm molecule, used as a capping layer on the surface of Ag NPs. The notable influence of OAm is tentatively linked to its structural and lipophilic resemblance to linoleic acid, whose capacity to reduce the interaction between the spike protein of the virus and the human ACE2 receptor has been proposed. Our findings indicate that the capping layer of the nanostructure is a crucial factor to be considered in the development of a new generation of smart materials against SARS-CoV-2 and possibly other viruses/emerging infectious agents.

## Supporting Information Available

Pictures of functionalized discs before and after contact with water, EDS analysis of the discs, UV–vis spectrum of Ag@OAc, IR spectra of Ag@OAc, Ag@OAm and the ligands, TGA analysis of Ag@OAm, and cytotoxicity of free OAm (PDF)
