# Supplementary material for: Silver Nanoparticles-Functionalized Textile against SARS-CoV-2: Antiviral Activity of the Capping Oleylamine Molecule
Source: ACS Appl Mater Interfaces. 2025 Jan 14;17(4):5710–8. doi: 10.1021/acsami.4c15289 (PMC11788990; doi:10.1021/acsami.4c15289)
Supplement: Supplementary file 1 — am4c15289_si_001.pdf [file am4c15289_si_001.pdf]

**Supporting information**

# Silver Nanoparticles Functionalized Textile Against SARS-CoV-2: Antiviral Activity of the Capping Oleylamine Molecule

*Tamyres Bernardo de Souza,*[a] *Alice S. Rosa*[b,c], *Pamella Constantino-Teles*[b,c], *Vivian Neuza S. Ferreira*[b], *Braulio S. Archanjo* [d,] *Carlos A. G. Soares,*[e] *Paulo H. S. Picciani*[f], *Rafael A. Allão Cassaro*[a*], *Milene Dias Miranda,*[b,c*] *Giordano Poneti,* [g,a,*]

[a]*Instituto de Química, Universidade Federal do Rio de Janeiro, Rio de Janeiro, RJ, 21941-909, Brazil*

[b] *Laboratory of Morphology and Virus Morphogenesis, Oswaldo Cruz Institute, Fiocruz, Avenida Brasil, Rio de Janeiro 21041-250, Brazil.*

[c]*Programa de pós-graduação em Biologia Celular e Molecular, Instituto Oswaldo Cruz, Fundação Oswaldo Cruz, Rio de Janeiro 21041-250, Brazil*

[d]*Materials Metrology Division, National Institute of Metrology, Quality, and Technology, Duque de Caxias, Rio de Janeiro 25250−020, Brazil*

[e]*Departamento de Genética, Universidade Federal do Rio de Janeiro, Rio de Janeiro, RJ,*

*21941-617, Brazil*

[f]*Instituto de Macromoléculas Professora Eloisa Mano, Universidade Federal do Rio de Janeiro, Rio de Janeiro, RJ, 21941-598, Brazil*

[g]*Dipartimento di Scienze Ecologiche e Biologiche, Università degli Studi della Tuscia, Largo dell'Università, 01100, Viterbo, Italy*

- Corresponding authors: allao.cassaro@iq.ufrj.br, mmiranda@ioc.fiocruz.br, giordano.poneti@unitus.it

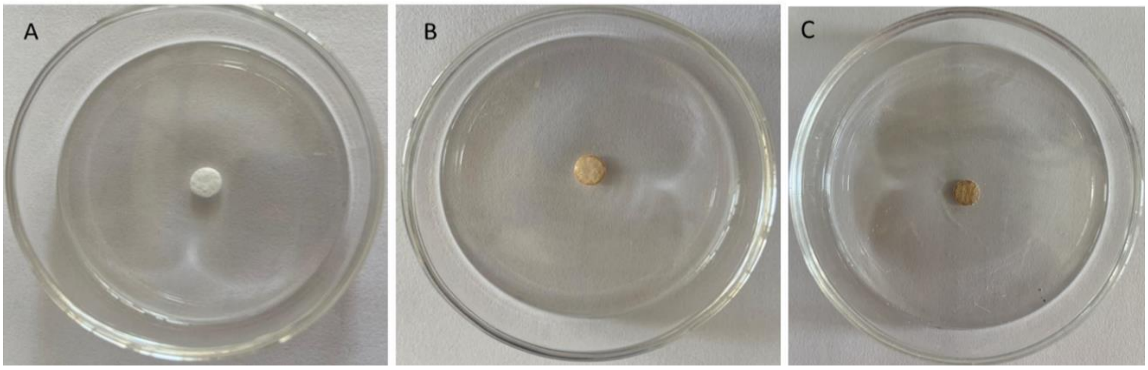


**Figure S1**: Pictures of disc of textile: disc (A), disc impregnated with Ag@OAm (B) and impregnated disc after 4 cycles of contact with water (C).

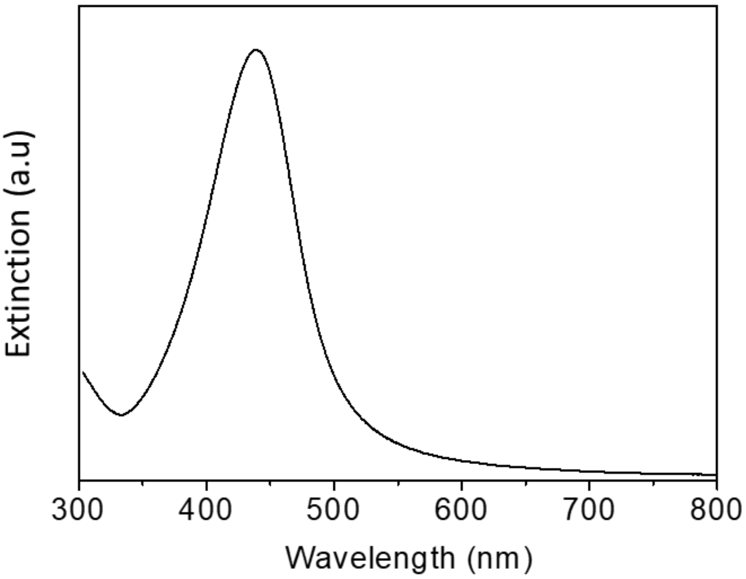


**Figure S2**: UV-Vis spectrum of Ag@OAc in toluene.

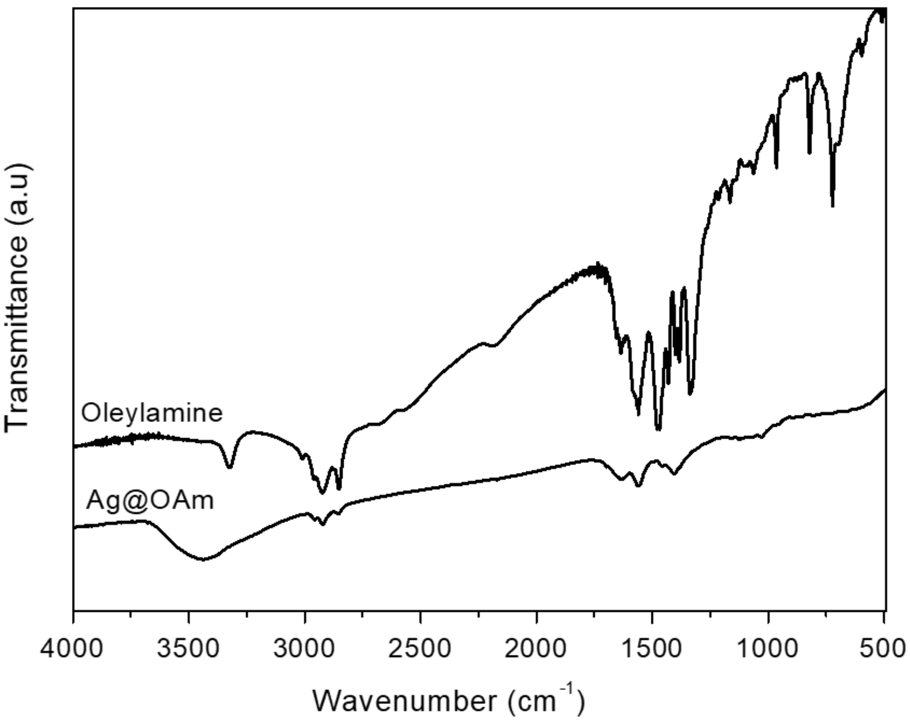


**Figure S3**: Infrared spectra of oleylamine and Ag@OAm.

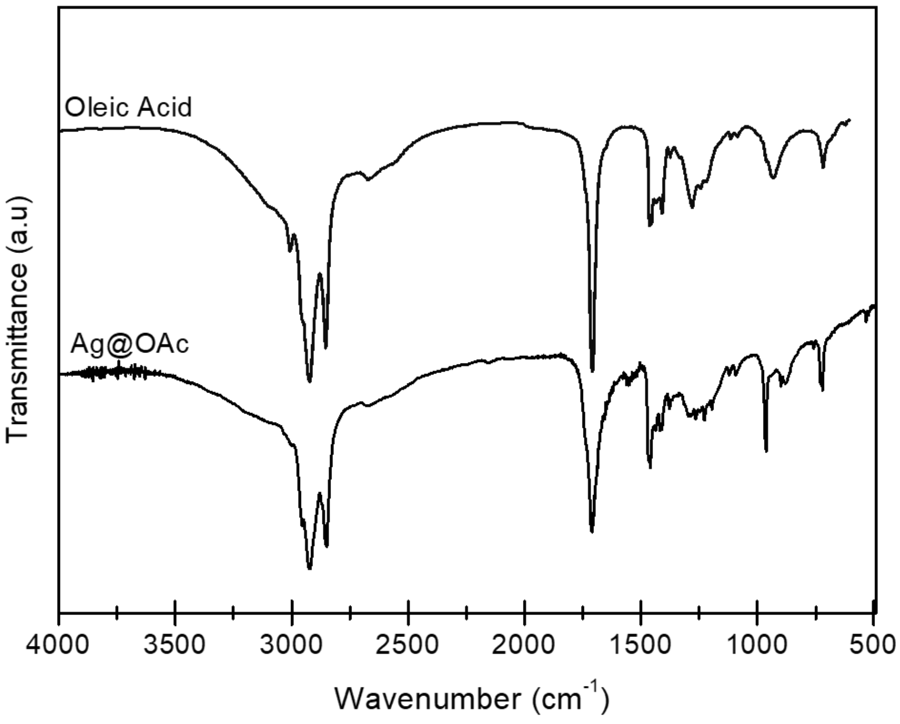


**Figure S4**: Infrared spectra of oleic acid and Ag@OAc.

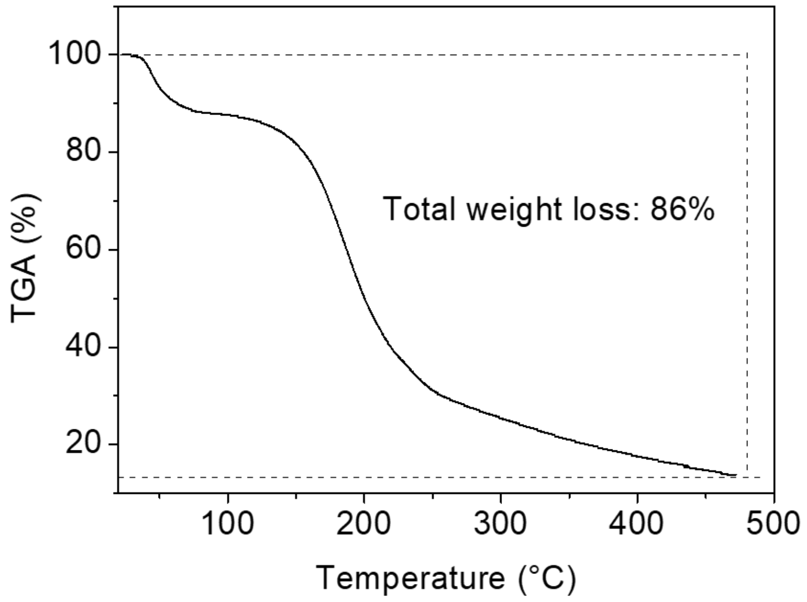


**Figure S5**: TGA results for Ag@OAm

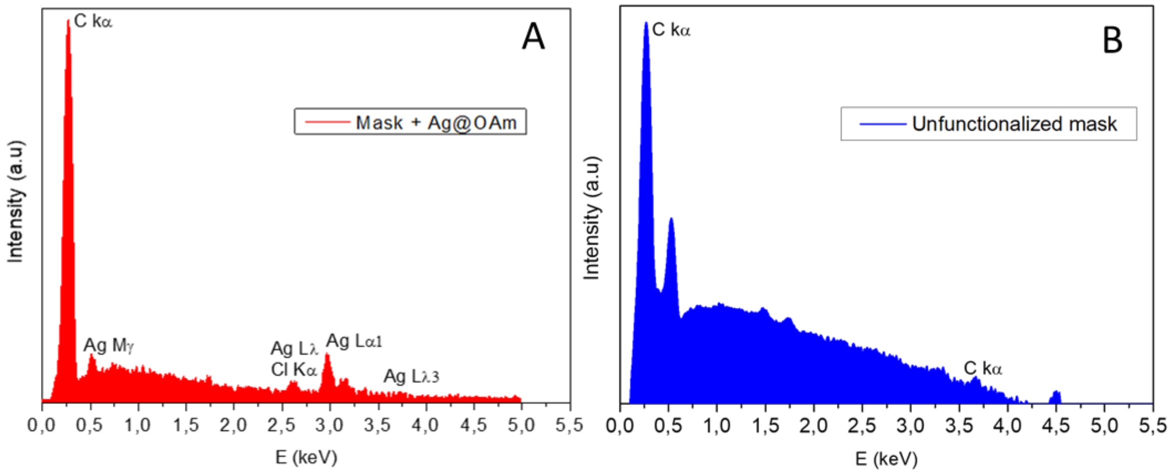


**Figure S6:** EDS analysis of textile functionalized with Ag@OAm (A) and unfunctionalized (B).

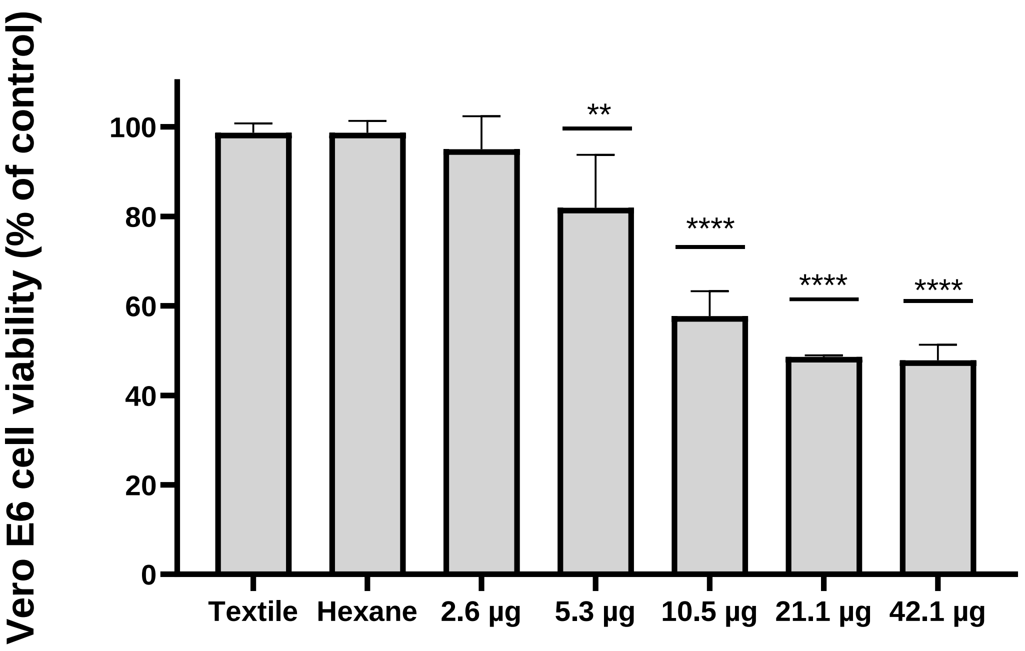


**Figure S7** – Cytotoxicity of oleylamine on Vero E6 cells. The cells were exposed to the culture medium after contact with functionalized textile with different amounts of oleylamine for 10 minutes (2.6, 5.3, 10.5, 21.1, 42.1 µg). The cell viability was assessed by MTT assay. *n*-hexane added to textile, as well as the pure textile were used as control. $n$=3, **$p$≤0.01, ****$p$≤0.0001.
